# Role of graphene quantum dots with discrete band gaps on SnO_2_ nanodomes for NO_2_ gas sensors with an ultralow detection limit†

**DOI:** 10.1039/d2na00925k

**Published:** 2023-04-28

**Authors:** Jinho Lee, Minsu Park, Young Geun Song, Donghwi Cho, Kwangjae Lee, Young-Seok Shim, Seokwoo Jeon

**Affiliations:** a Department of Materials Science and Engineering, Korea Advanced Institute of Science and Technology (KAIST) Daejeon 34141 Republic of Korea Jeon39@korea.ac.kr; b Querrey Simpson Institute for Bioelectronics, Northwestern University Evanston IL 60208 USA; c Electronic Materials Research Center, Korea Institute of Science and Technology (KIST) Seoul 02791 Republic of Korea; d Thin Film Materials Research Center, Korea Research Institute of Chemical Technology Yuseong Daejeon 34114 Republic of Korea; e Department of Information Security Engineering, Sangmyung University Cheonan 31066 Republic of Korea; f School of Energy, Materials and Chemical Engineering, Korea University of Technology and Education Cheonan 31253 Republic of Korea; g Department of Materials Science and Engineering, Korea University Seoul 02841 Republic of Korea

## Abstract

NO_2_ is a major air pollutant that should be monitored due to its harmful effects on the environment and human health. Semiconducting metal oxide-based gas sensors have been widely explored owing to their superior sensitivity towards NO_2_, but their high operating temperature (>200 °C) and low selectivity still limit their practical use in sensor devices. In this study, we decorated graphene quantum dots (GQDs) with discrete band gaps onto tin oxide nanodomes (GQD@SnO_2_ nanodomes), enabling room temperature (RT) sensing towards 5 ppm NO_2_ gas with a noticeable response ((*R*_a_/*R*_g_) − 1 = 4.8), which cannot be matched using pristine SnO_2_ nanodomes. In addition, the GQD@SnO_2_ nanodome based gas sensor shows an extremely low detection limit of 1.1 ppb and high selectivity compared to other pollutant gases (H_2_S, CO, C_7_H_8_, NH_3_, and CH_3_COCH_3_). The oxygen functional groups in GQDs specifically enhance NO_2_ accessibility by increasing the adsorption energy. Strong electron transfer from SnO_2_ to GQDs widens the electron depletion layer at SnO_2_, thereby improving the gas response over a broad temperature range (RT–150 °C). This result provides a basic perspective for utilizing zero-dimensional GQDs in high-performance gas sensors operating over a wide range of temperatures.

## Abstract 2

The decoration of graphene quantum dots enhances the NO_2_ gas sensing performance of SnO_2_ nanodomes by utilizing both electrical and chemical sensitization effects, leading to an impressive reduction in the theoretical detection limit.

## Graphical abstract

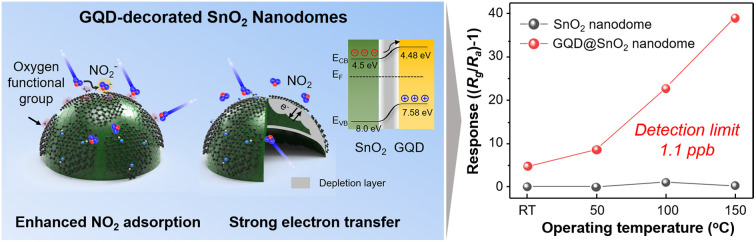

## 1. Introduction

Nitrogen dioxide (NO_2_), generated by automobile exhausts and fossil fuel combustion, is one of the main causes of air pollution. Atmospheric NO_2_ leads to harmful effects not only on the environment but also on human health by causing respiratory diseases.^1^ The U.S. Environmental Protection Agency (US EPA) has announced that the national ambient air quality standard for NO_2_ levels in the atmosphere is 53 ppb. Therefore, it is of great importance to accurately detect the low concentration NO_2_ gas with extremely high sensitivity.

Over the past few decades, chemiresistive-type gas sensors built using semiconductor metal oxides (SMOs), such as SnO_2_, WO_3_, In_2_O_3_, Nb_2_O_5_, TiO_2_, NiO, and ZnO, have received much attention due to their incomparable advantages including low cost, good durability, easy fabrication, and high sensitivity compared with other types of gas sensors.^2,3^ However, poor selectivity towards various gases, a chronic problem of SMO gas sensors, interrupts the discrimination of gases in the atmosphere, and thus, ultimately hinders the practical application of SMO gas sensors. Furthermore, the good sensing capability of SMO gas sensors is valid usually at high temperatures (>200 °C), which leads to difficult integration with other devices and is not ideal for Internet of Things (IoT) applications.^4^

To moderate the working conditions and improve gas selectivity, catalytic materials that enhance the adsorption of a gas molecule can be functionalized on the surface of metal oxides. Various functionalization strategies, along with the decoration of graphene-based nanomaterials, have been employed due to the large specific surface area, good charge transport properties, and specific catalytic effects of functional groups.^5,6^ However, the large lateral size of pristine graphene, synthesized by a conventional method such as chemical vapor deposition and exfoliation, inhibits the access of gas molecules. Additionally, the low electrical resistance of graphene shorts the electric current, which hides the resistance changes in metal oxides. These issues associated with the use of graphitic materials can be addressed by reducing the size of graphene to the nanometer size, and the resultant materials are called graphene quantum dots (GQDs). The GQDs defined in this study are synthesized by non-acidic exfoliation with graphite intercalation compounds (GICs), resulting in unique luminescent properties and nanomorphology.^7–21^ This non-acidic synthesis process conserves 2D flat geometry of sp^2^ carbon in GQDs, which is distinct from that of previously reported ‘carbon nanodots’ that is a complex of sp^2^–sp^3^ carbon.^13,22,23^

GQDs, a nanometer-sized family of graphene, have the advantage of a large surface area due to their very small size, which can contribute to an improved gas response *via* a significant increase in the number of active sites. In addition, GQDs can have discrete electronic band structures mostly due to subdomains that only appear within GQDs under controlled oxidation.^8^ These subdomains are composed of several sp^2^ carbon hexagons that are confined and formed during the attachment of oxygen functional groups to graphene. π-electrons, which were initially delocalized in the basal plane of graphene, become localized within these small sp^2^ clusters. This creates a discrete band gap of π–π* intrinsic states. Consequently, the quantum confinement effect of GQDs applies only to these sp^2^ subdomains and not the entire region of GQDs, which is unlike traditional semiconductor QDs.^13^ These electronic band structures enable effective charge transfer and charge separation at the interface of metal oxides. The excellent charge transfer effects of GQDs to TiO_2_ through the suitable band structure of GQDs have been presented in previous intense research by our group.^10,21^ Recent studies have shown that nitrogen-doped GQDs (N-GQDs) improve the NO_2_-sensing performance of SnO_2_ by increasing the electron transfer/space charge modulation depth and NO_2_ adsorption sites.^24^ The zero-dimensional (0D) heterostructure of N-GQD/SnO_2_ quantum dots exhibits an enhanced response (*R*_g_/*R*_a_ = 4336) towards 100 ppb at 50 °C. The zero-/three-dimensional (0D/3D) heterostructure of a N-GQD/mesoporous SnO_2_ hollow cube shows an improved response (*R*_g_/*R*_a_ = 417) towards 1 ppm NO_2_.^25^ However, heteroatom doping of GQDs is normally performed in harsh environments, such as high-temperature treatment and acidic treatment, which significantly degrades the quality of GQDs. Moreover, heteroatom doping impairs the sp^2^ hybridization of carbon into sp^3^ hybridization, leading to the loss of the characteristic feature of graphene. These complex and uncontrolled structures lead to difficulty in understanding and utilizing the advantages of two-dimensional graphitic materials in SMO gas sensors. Therefore, the investigation of GQDs with a highly preserved sp^2^ domain is important to provide an essential background for graphene-functionalized gas sensors.

In this study, we present GQD-decorated SnO_2_ (GQD@SnO_2_) nanodomes for a highly efficient NO_2_ gas sensor using GQDs with discrete band gaps. A highly ordered SnO_2_ nanodome array is used to realize a large active area and well-defined potential barrier, resulting in an improvement of the gas response and recovery time.^26,27^ By decorating 5 nm-sized GQDs onto the surface of SnO_2_ nanodomes, the response to 5 ppm NO_2_ is significantly enhanced compared to pristine SnO_2_ nanodomes at room temperature, 50 °C, 100 °C, and 150 °C, with an ∼118-fold response enhancement at an operating temperature of 150 °C. The role of GQDs on SnO_2_ nanodomes was systematically investigated by the change in electrical properties and chemical bonding states. The GQDs with controlled oxygen functional groups for realizing discrete band gaps are closely bound to the surface of SnO_2_ nanodomes and increase the adsorption energy of NO_2_ gases at room temperature. Highly efficient electron transfer from SnO_2_ to GQDs enlarges the electron depletion layer of SnO_2_ nanodomes, which enables NO_2_ gas sensing at room temperature with high gas response.

## 2. Materials and methods

### 2.1 Fabrication of GQD@SnO_2_ nanodomes

Pt/Ti (thickness of 150 nm/30 nm) interdigitated electrode patterns (IDEs) consisting of 20 electrodes were fabricated on a SiO_2_/Si substrate (thickness of 300 nm/550 μm). The distance between each electrode is 5 μm, and the active sensing area is 0.8 mm × 0.8 mm. Nanodome-like structures were fabricated by the soft-templating method.^28^ Polystyrene (PS) beads (700 nm, 5.0 wt%, Spherotech, USA) were dispersed in a water : ethanol = 1 : 1 (v/v) solution by a centrifuge process after the concentration reached 10 wt%. The PS bead solution was pipetted onto a glass slide positioned at an angle of 45° in a Petri dish with deionized water. The Pt/Ti IDE patterned substrates and slide glass were treated by O_2_-plasma treatment (CUTEMP, femtoscience) for 10 minutes before fabrication. The pipetted solution was dispersed onto the surface of deionized water and allowed to form a PS bead monolayer. The Pt/Ti IDE patterned substrates were dipped into water and the PS bead monolayer was pulled out. Then, the PS bead monolayer was dried at room temperature for 24 hours. SnO_2_ was deposited onto the PS bead monolayer with masking tape by using an electron-beam evaporator. A 150 nm thick SnO_2_ layer was deposited at a rate of 1 Å s^−1^. The SnO_2_ nanodomes on the substrates were annealed at 500 °C for 1 hour to simultaneously remove the PS templates and crystallize the SnO_2_ nanodomes.

The GQDs were prepared from graphite intercalation compounds (GICs) through a previous method.^15^ First, graphite and potassium sodium tartrate (KNaC_4_H_4_O_6_·4H_2_O) were vigorously mixed at a ratio of 1 : 15 (w/w) and then ground. The mixture was heated in a heating mantle at 250 °C for 24 hours, which led to the formation of GICs. The as-prepared GICs were immersed in DI water and sonicated to exfoliate and cut the graphite. The crude GQD solution was filtered using centrifugal microfilters (10 000 NMWL, Amicon Ultra-15), followed by dialysis using a dialysis membrane for 3 days to remove any impurities and obtain pure GQDs <5 nm in size. The GQD solution (0.1 mg ml^−1^) was drop cast (10 drops) onto SnO_2_ nanodomes and allowed to dry at room temperature for 24 hours.

### 2.2 Characterization and gas response measurements

The morphology of the GQD@SnO_2_ nanodomes was investigated by field-emission scanning electron microscopy (FE-SEM, SU 5000, Hitachi). The structures and fast Fourier transform (FFT) images of GQDs were investigated by transmission electron microscopy (TEM, Tecnai F20, FEI Company). The crystallinity of the sensors was measured by X-ray diffraction (XRD, Ultima IV, RIGAKU) with a Cu-Kα radiation source (wavelength 1.5418 Å). The chemical bonding and binding energies of the sensor materials were investigated by X-ray photoelectron spectroscopy (XPS) using a K-alpha system (Thermo VG Scientific) with an Al-Kα X-ray source. The Raman spectra of GQDs on SnO_2_ nanodomes were collected using a Senterra system (Bruker) with a 532 nm laser. The samples for XPS analysis and Raman analysis were prepared by annealing for 1 hour on a hot plate at room temperature, 50 °C, 100 °C, and 150 °C. The oxygen content in the GQDs was estimated using Auger electron spectroscopy (AES) with a source electron beam energy of >10 kV.

The responses of target gases were measured in a quartz tube with a 1-inch furnace (Lindberg, blue M). The operating temperature was controlled by a 1-inch furnace at room temperature, 50 °C, 100 °C, and 150 °C to evaluate the gas response mechanism at different operating temperatures. The gas flows were controlled to give a constant flow rate of 1000 sccm under dry conditions (RH 0) using a mass-flow controller. The sensor resistance was measured using a Keithley 2401 instrument with a DC bias voltage of 0.5 V.

## 3. Results and discussion

### 3.1 Morphological and structural characterization of GQD@SnO_2_ nanodomes

**Fig. 1:**
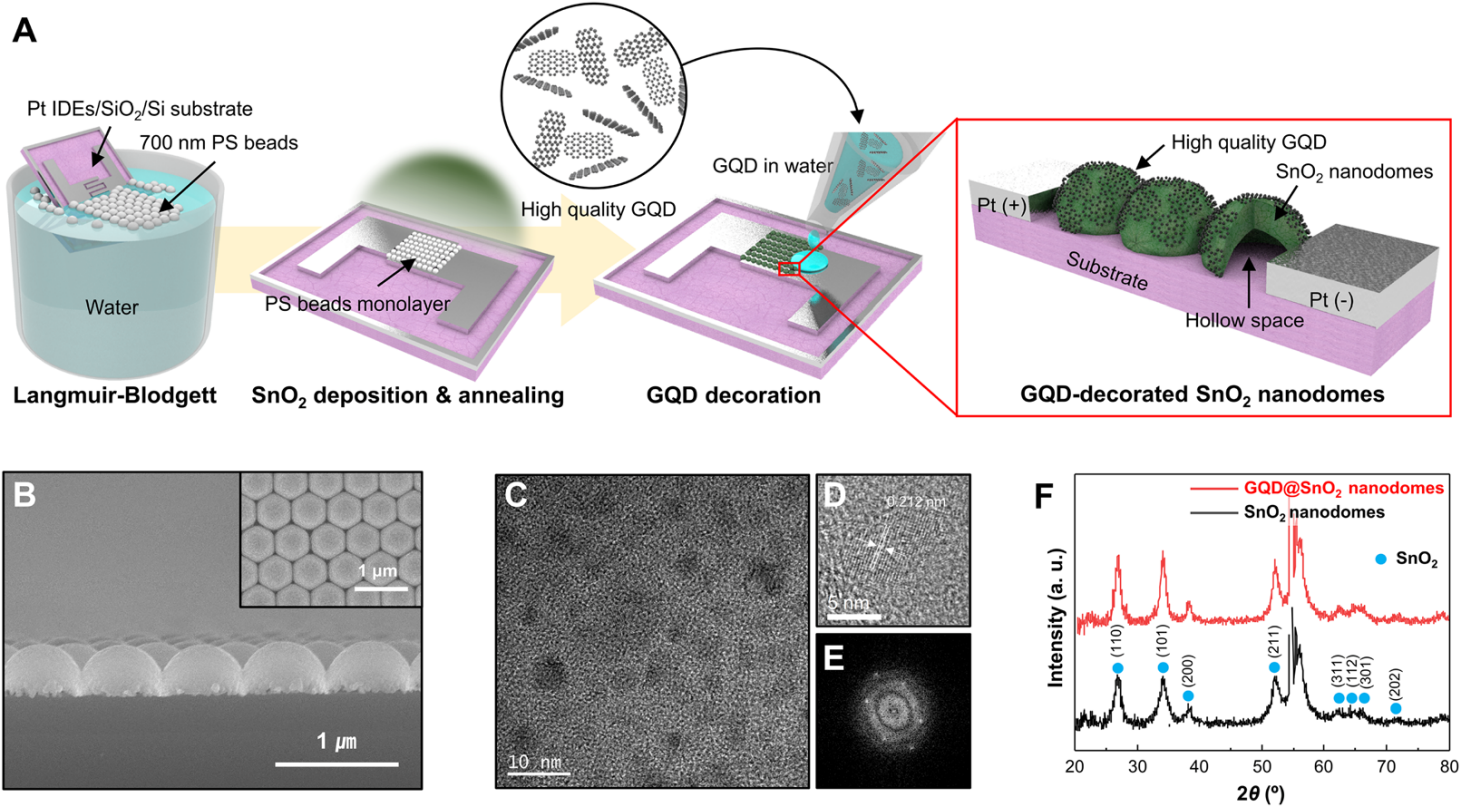
Fabrication and characterization of GQD-decorated SnO_2_ (GQD@SnO_2_). (A) Schematic illustration of the fabrication process for a GQD@SnO_2_ nanodome based gas sensor. (B) Cross-sectional SEM image of GQD@SnO_2_ nanodomes. The inset shows a plain-view SEM image of GQD@SnO_2_ nanodomes. (C) HR-TEM image of GQD@SnO_2_ nanodomes, (D) lattice fringe images, and (E) FFT of the GQDs. (F) XRD patterns of SnO_2_ nanodomes and GQD@SnO_2_ nanodomes.

Fig. 1A shows a schematic diagram of the overall fabrication procedure for a GQD@SnO_2_ nanodome based gas sensor. Note that the structure of GQD@SnO_2_ nanodomes is illustrated with plane-to-surface decoration between the monolayer GQD plane and the SnO_2_ nanodomes for intuitive understanding, which might have other orientations such as edge-to-surface in the actual structure. In brief, a SnO_2_ thin film was deposited onto a PS bead monolayer by using an electron beam evaporator. During subsequent thermal treatment, the PS bead was removed and SnO_2_ thin films were crystallized simultaneously. Finally, the fabrication of a GQD@SnO_2_ nanodome gas sensor was completed by drop-casting GQD solution and drying at room temperature. The GQDs fabricated from the GICs have an average diameter of 4.38 nm (Fig. S1†). They have controlled oxygen functional groups with a discrete band gap^8,10,12^ and have a low oxygen content of 3.91 at%, as measured by AES analysis (Fig. S2†).

Fig. 1B shows the morphology of GQD@SnO_2_ nanodomes observed using SEM micrographs. The SnO_2_ nanodomes are in contact with adjacent nanodomes as a single layer and show highly ordered, hexagonal close-packed structures. A cross-sectional SEM micrograph clearly shows that the SnO_2_ nanodomes form a perfect monolayer (Fig. 1B). The microstructure and crystallinity of the GQD@SnO_2_ nanodomes were characterized by HR-TEM and XRD. The SnO_2_ nanodomes consist of nanocrystallites with a grain size of 30–40 nm, and the HR-TEM image shows a lattice spacing of 0.33 nm for the (110) plane (Fig. S3†). As shown in Fig. 1C–E, the HR-TEM image and the corresponding fast Fourier transform (FFT) pattern of the GQDs prove that the graphitic structure has a lattice spacing of 0.212 nm and a hexagonal structure, respectively.^9^ The crystallinity of the SnO_2_ nanodomes was investigated by XRD (Fig. 1F). The presence of multiple peaks indicates that the SnO_2_ nanodomes are polycrystalline, which corresponds to rutile SnO_2_ (JCPDS no. 01-070-4117). It was difficult to observe the characteristic peaks for GQDs, which implies that the GQDs are deposited as a single layer without stacking.

### 3.2 Gas sensing mechanism of metal oxide gas sensors

The working principle for metal oxide gas sensors is based on the modulation of an electron depletion layer. For n-type metal oxides such as SnO_2_, the oxygen in the air is adsorbed onto the metal oxide surface in the form of ion states by withdrawing electrons from the metal oxide, which induces the formation of an initial electron depletion layer at the surface of the metal oxide.^29,30^ Depending on the type of target gas, the target either gas reacts with surface oxygen ions to release electrons (reducing gas) to the metal oxide or extracts electrons (oxidizing gas) from the metal oxide, leading to changes in the width of the electron depletion layer. The gas response is calculated using the electrical resistance of the gas sensor under an air flow and the target gas, which is defined as *R*_a_ and *R*_g_, respectively. NO_2_ gas is a representative oxidizing gas that extracts electrons from metal oxides and is adsorbed on the surface as NO_2_^−^(ads), increasing the electrical resistance of the sensor. Accordingly, the gas response (S) is calculated in the form of ((*R*_g_/*R*_a_) − 1). The response to NO_2_ gas is related to the amount of absorbed oxygen ions on the metal oxide. As the operating temperature increases, the adsorption of oxygen ions and NO_2_ occurs easily due to high thermal energy. The suggested gas reaction pathways for oxygen ions and NO_2_ gas are summarized as follows:^29,31,32^

1 O_2_(gas) + e^−^ ↔ O_2_^−^(ads)

2 O_2_^−^(ads) + e^−^ ↔ O_2_^2−^(ads) ↔ 2O^−^(ads)

3 NO_2_(gas) + Sn^2+^ ↔ NO_2_^−^(ads) + Sn^3+^

4 NO_2_(gas) + O_2_^−^(ads) + 2e^−^ ↔ NO_2_^−^(ads) + 2O^−^(ads)

### 3.3 NO_2_ gas sensing performance of GQD@SnO_2_

**Fig. 2:**
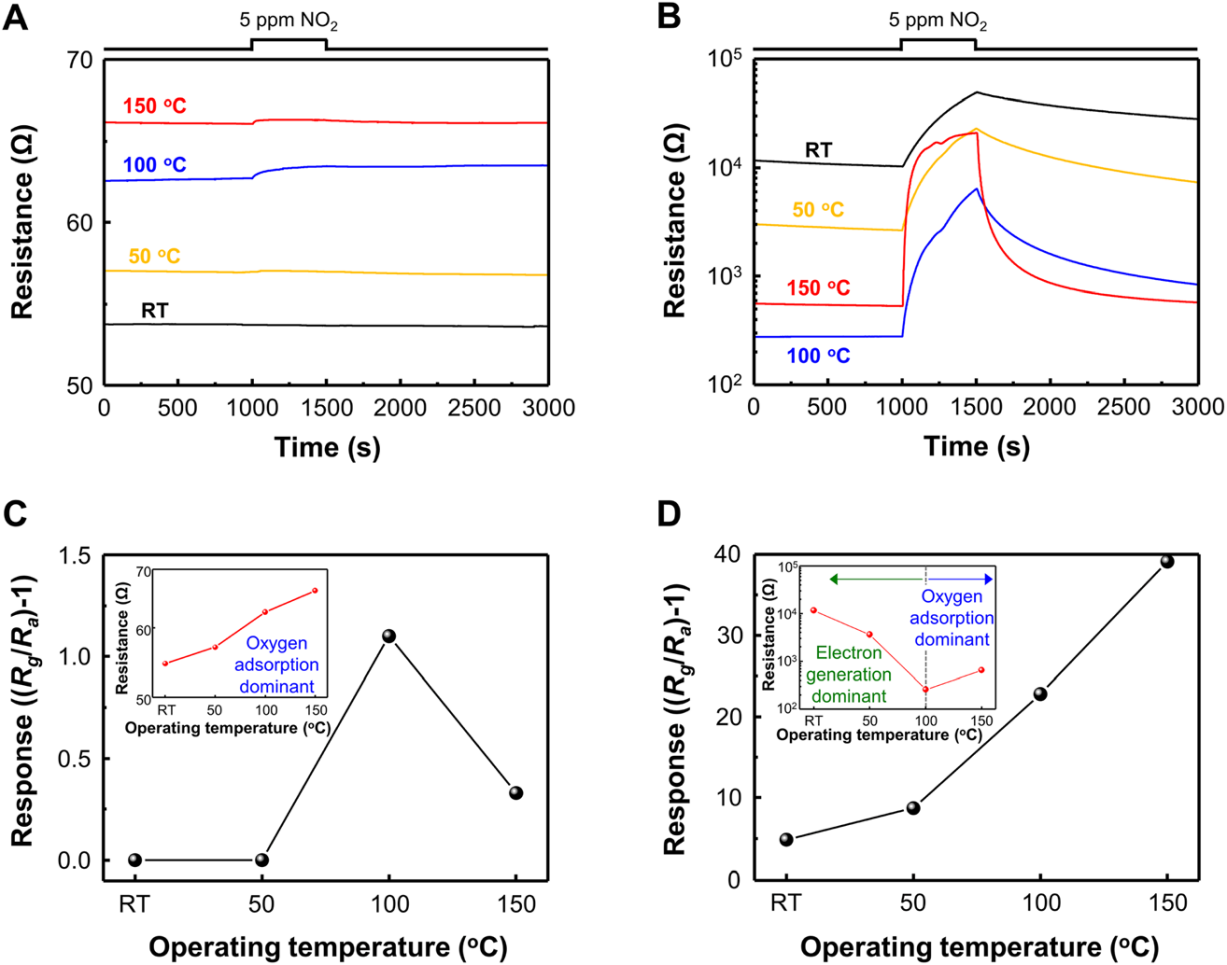
Resistance curves for 5 ppm NO_2_ as a function of operating temperature for (A) pristine SnO_2_ nanodomes and (B) a GQD@SnO_2_ nanodome based gas sensor. Gas response for (C) pristine SnO_2_ nanodomes and (D) a GQD@SnO_2_ nanodome based gas sensor. The insets of (C and D) represent the base resistance as a function of operating temperature.

Based on the gas sensing mechanism of metal oxide, the response of pristine SnO_2_ and GQD@SnO_2_ nanodomes to 5 ppm of NO_2_ was measured with an operating temperature gradient from RT (27 °C) to 150 °C (Fig. 2). For the low operating temperature range (RT, 50 °C), the pristine SnO_2_ nanodomes exhibit no gas response since oxygen ions are poorly generated to adsorb NO_2_ gases. The pristine SnO_2_ nanodomes can detect NO_2_ gases at a temperature of over 100 °C with a gas response value of 1.1 (Fig. 2A). Interestingly, the response to 5 ppm NO_2_ is measurable even at room temperature after GQD decoration onto SnO_2_ nanodomes, which implies that the GQDs have the capability to enhance NO_2_ adsorption on the SnO_2_ surface (Fig. 2B). The responses to 5 ppm NO_2_ and base resistance changes as a function of operating temperature for each gas sensor are summarized in Fig. 2C and D. The response value for GQD@SnO_2_ nanodomes to 5 ppm NO_2_ at an operating temperature of RT, 50 °C, 100 °C, and 150 °C is 4.80, 8.70, 22.8, and 39.1, respectively, which are much higher than those for pristine SnO_2_ nanodomes in all temperature ranges. Moreover, the gas response for GQD@SnO_2_ nanodomes increases as the operating temperature increases, and the resulting gas response/recovery times for GQD@SnO_2_ nanodomes at 150 °C are 322 s/105 s, respectively (Table S2†). Compared to bare SnO_2_ nanodomes at 150 °C, GQD decoration improves the recovery time (bare SnO_2_ nanodomes: 1247 s). However, under humid conditions with a relative humidity (RH) of 50%, the gas responses of GQD@SnO_2_ nanodomes decrease (Fig. S4†), which might be ascribed to high affinity of GQDs to moisture that interrupts the access of the NO_2_ molecule to the active adsorption site of GQD@SnO_2_ nanodomes.^33^

As the operating temperature increases, the base resistance of pristine SnO_2_ nanodomes increases due to increments in the amount of adsorbed oxygen ions on the surface but remains lower than 70 Ω even at 150 °C (inset, Fig. 2C). The result of current–voltage (*I*–*V*) measurement shows that the base resistance of GQD@SnO_2_ is higher than that of pristine SnO_2_ nanodomes in ambient air at room temperature (Fig. S5†). This indicates that the GQDs enlarge the electron depletion layers on the SnO_2_ surface, resulting in an increased base resistance above 104 Ω (inset, Fig. 2D). Until the operating temperature reaches 100 °C, the electron generation effect dominates the change in electrical resistance as the GQDs spatially hinder the access of oxygen to the SnO_2_ surface. The oxygen adsorption effects become dominant at temperatures over 150 °C, assisted by high thermal energy. These results with the expansion of the electron depletion layer indicate that there is a strong charge transfer interaction between GQDs and SnO_2_ nanodomes, which suggests that the GQD@SnO_2_ nanodomes can be used as a high response gas sensor for 5 ppm NO_2_ at low operating temperature.^10,21^

**Fig. 3:**
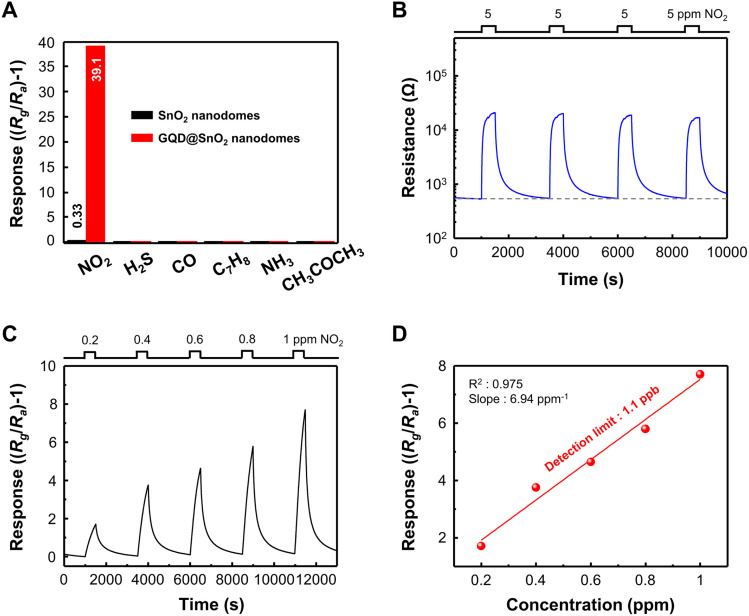
(A) Selective NO_2_ gas sensing performance of GQD@SnO_2_ nanodome based gas sensors. (B) Resistance curve of GQD@SnO_2_ to 5 ppm NO_2_ with repeated exposure. (C) Response curves to different NO_2_ concentrations under a 1 ppm concentration. (D) Linear fit of the responses as a function of NO_2_ concentration.

Fig. 3A shows the sensor responses to various pollutant gases (NO_2_, H_2_S, CO, C_7_H_8_, NH_3_, and CH_3_COCH_3_) for verifying the selectivity of GQD@SnO_2_ nanodomes. The GQD@SnO_2_ gas sensor exhibits the highest response to 5 ppm NO_2_. On the other hand, the GQD@SnO_2_ gas sensor does not show any gas response to the other gases (50 ppm H_2_S, CO, C_7_H_8_, NH_3_, and CH_3_COCH_3_). These results are attributed to NO_2_ gas being an oxidizing gas, which can release electrons from the SnO_2_ surface by itself or with oxygen ions; however, the other gases are reducing gases, which should react with surface oxygen ions to release electrons. Therefore, decoration of GQDs leads to no improvement in the gas response to reducing gases, but rather reduces the gas responses, due to decreased oxygen ion adsorption on the SnO_2_ surface, as mentioned in the base resistance analysis.

We repeatedly exposed the GQD@SnO_2_ nanodomes to 5 ppm NO_2_, as shown in Fig. 3B. The base resistance is maintained after several adsorption and desorption cycles of NO_2_ gas, which means that the gas sensor can be completely recovered to its initial state. GQD@SnO_2_ nanodomes were exposed to extremely low concentrations of NO_2_ ranging from 0.2 ppm to 1 ppm at optimal temperature, as shown in Fig. 3C. The GQD@SnO_2_ nanodomes reveal a clear gas response even at 0.2 ppm NO_2_, and the gas response shows a linear relationship with the gas concentration (slope = 6.94 ppm^−1^, *R*^2^ = 0.975). Moreover, the calculated theoretical detection limit as shown in Fig. 3D is 1.1 ppb, which is the lowest value obtained compared to previously reported NO_2_ gas sensors that use metal oxide/graphene-based nanostructures, as summarized in Table S1.† Low oxidized GQDs with highly preserved sp^2^ carbon structures can be decorated on the SnO_2_ nanodomes with high density. This enables highly sensitive NO_2_ sensing with an ultralow detection limit, and details on the role of GQDs will be presented in the next section.

### 3.4 Surface analysis of GQD@SnO_2_ nanodomes

**Fig. 4:**
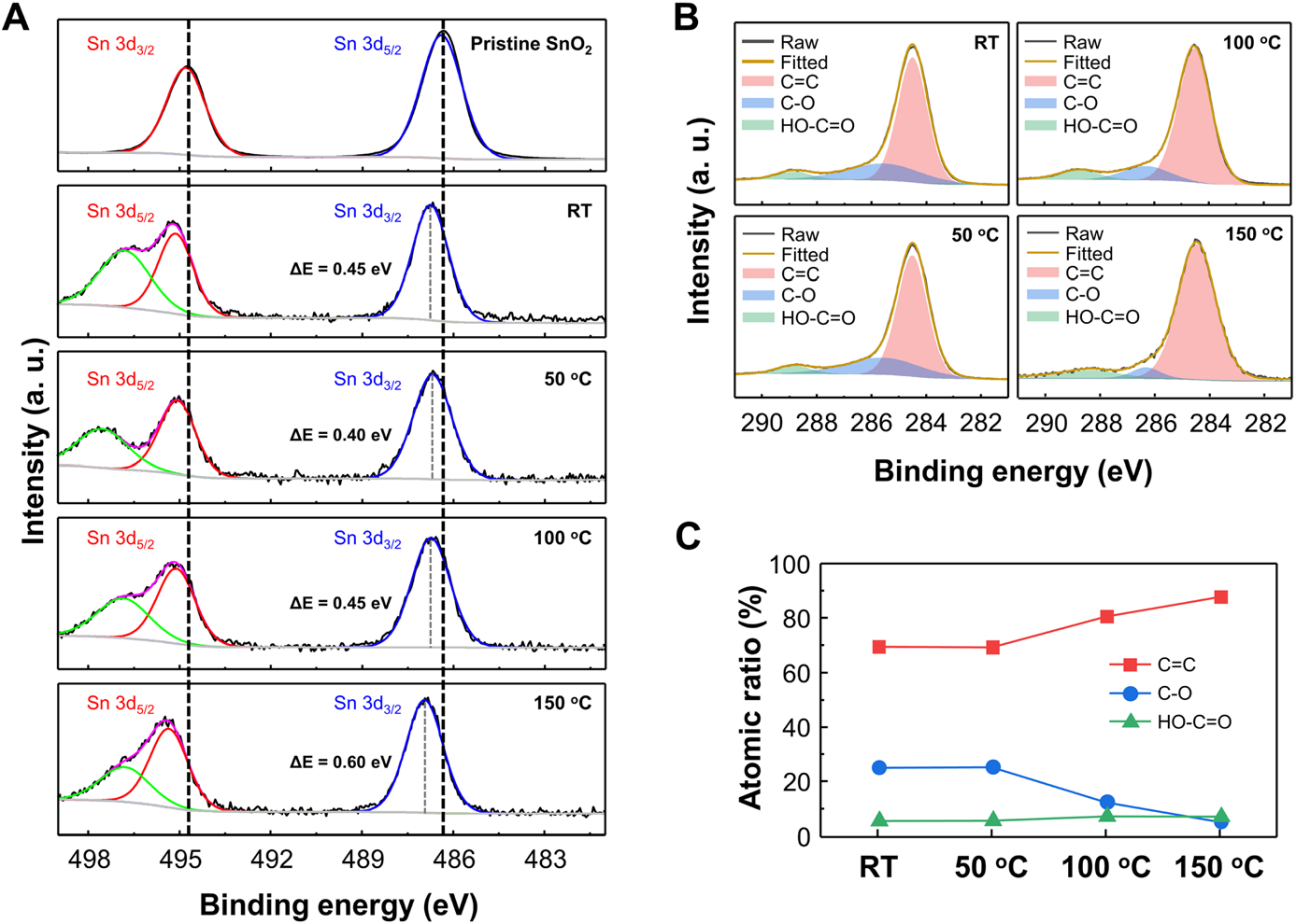
X-ray photoelectron spectroscopy (XPS) (A) Sn 3d and (B) C 1s spectra of pristine SnO_2_ nanodomes and GQD@SnO_2_ nanodomes prepared at various annealing temperatures. (C) Atomic ratio of carbon bonds as a function of annealing temperature.

To clarify the mechanism for electron depletion layer formation and gas sensing enhancement due to GQD decoration, we examined the surface compositions and corresponding atomic states of GQD@SnO_2_ nanodomes by XPS analysis (Fig. 4). Fig. 4A displays the Sn 3d spectra of pristine SnO_2_ nanodomes and GQD@SnO_2_ nanodomes with different annealing temperatures. The peaks at 486.3, 494.8, and 496.8 eV are assigned to the Sn 3d_5/2_, Sn 3d_3/2_, and Sn (loss) peaks, respectively.^34,35^ For the GQDs@SnO_2_ nanodomes at room temperature without an annealing process, the binding energy of Sn 3d shifts by as much as 0.45 eV to a higher value. The binding energy shift towards a higher value indicates an electron transfer from SnO_2_ to GQDs.^26^ These results are consistent with those of the base resistance analysis, in which the widening of the electron depletion layer increases the electrical resistance. Until the annealing temperature reaches 100 °C, the magnitude of the binding energy shift is similar to that observed at room temperature. On the other hand, after annealing at 150 °C, the binding energy shift increases to 0.6 eV with an intense electron transfer phenomenon. The larger binding energy shift leads to a further widening of the electron depletion layer with an increased NO_2_ gas response.

Fig. 4B and C show the C 1s spectra and carbon bonding atomic ratio for GQD@SnO_2_ nanodomes. The C 1s spectra show peaks at 284.5 eV (C=C), 285.8 eV (C–O), and 288.8 eV (HO–C=O) at overall annealing temperatures, which are characteristic bonds in the GQDs. The high C=C ratio (69.4 at%) for GQDs at room temperature demonstrates the highly preserved sp^2^ domain in GQDs synthesized by the GIC method. As the annealing temperature increases, the number of oxygen functional groups decreases due to thermal reduction, while the C=C bond is restored (Fig. 4C). The restoration of the sp^2^ C=C bond of GQDs is also confirmed by Raman spectrum analysis (Fig. S6†). In the Raman spectrum of the GQDs@SnO_2_ nanodomes, a disorder (D) band at 1393 cm^−1^ and a sp^2^ carbon (G) band at 1591 cm^−1^ clearly appear and the *I*_D_/*I*_G_ ratio decreases as the annealing temperature increases, which represent the increments of the sp^2^ carbon structure in GQDs. This can increase the delocalization of π-electrons relative to the unannealed GQDs, thereby enhancing the electron donating properties.^14,36^ This also indicates that the energy levels associated with defects that act as charge trapping sites also decrease relative to those of unannealed GQDs. Accordingly, electron transfer from the conduction band of SnO_2_ to the lowest unoccupied molecular orbital (LUMO) level of GQDs occurs more efficiently. This can widen the electron depletion layer on the SnO_2_ surface, which enables a higher NO_2_ gas response at a higher operating temperature (150 °C). These remarkable surface properties of the GQDs allow the GQD@SnO_2_ nanodome gas sensor to detect NO_2_ gas even at room temperature, where the pristine SnO_2_ nanodome gas sensor cannot do the same.

### 3.5 Role of GQDs on enhanced NO_2_ gas sensing performance

**Fig. 5:**
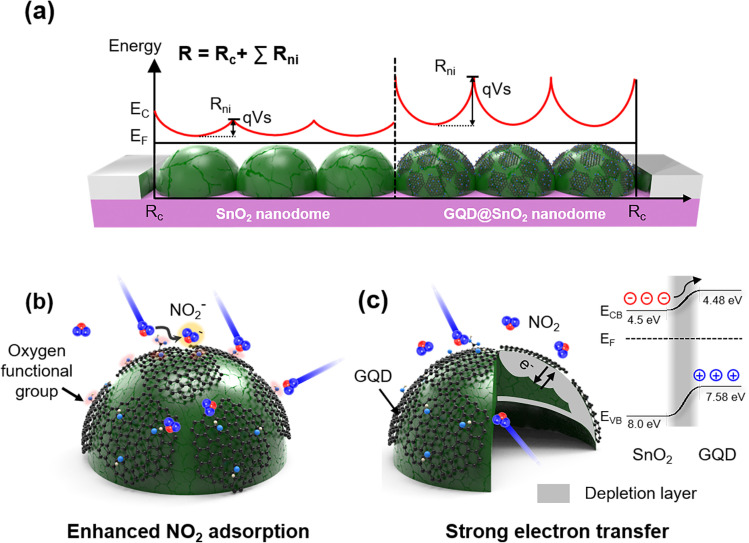
Schematic illustration for (A) initial potential barrier formation for the SnO_2_ nanodomes structure. (B and C) Schematic illustration of the NO_2_ sensing mechanism for GQD@SnO_2_ nanodomes showing (B) enhanced NO_2_ adsorption due to the GQDs and (C) formation of an electron depletion layer with its electronic band structure.

The enhancement mechanism for the GQD@SnO_2_ nanodome gas sensor is illustrated in Fig. 5. The well-defined potential barrier between nanodomes effectively modulates the electrical resistance to amplify the NO_2_ gas response (Fig. 5A). The total electrical resistance of the sensor includes the contact resistance (*R*_c_) with the electrodes and resistance of each nanodome (*R*_ni_), including the potential barrier between nanodomes (*E*_c_: conduction band, *E*_F_: Fermi level of SnO_2_, *q*: electron charge, and *V*_s_: barrier potential). In addition, the decoration of GQDs on SnO_2_ nanodomes increases the potential barrier between nanodomes *via* an enlargement of the electron depletion layer, which can amplify the gas response. Additionally, the 3-dimensional nanostructures enlarge the total surface area where the gas responds, resulting in an overall improvement in the gas response.

The role of GQD decoration on SnO_2_ is explained using the following two aspects (Fig. 5B and C): first, the GQDs enhance NO_2_ gas adsorption on the oxygen functional group by lowering the adsorption energy of NO_2_ on the SnO_2_ surface (Fig. 5B). As discussed above, the ease of NO_2_ adsorption on the SnO_2_ surface synergistically improves the gas response with better electron attraction for the NO_2_ gas. Density functional theory (DFT) studies show that the calculated adsorption energy of NO_2_ on a perfect SnO_2_-cassiterite (110) surface is approximately −0.52 eV, while that on hydroxyl groups on graphene is −0.91 eV.^37,38^ This implies that functional groups in graphene can induce a stronger interaction with NO_2_.^24,25,39–45^ Second, the GQDs widen the electron depletion layer and induce strong electron transfer (Fig. 5C). The flat 2D feature of GQDs facilitates close contact with the SnO_2_ surface, which enlarges the electron depletion layer, as observed in the base resistance analysis (Fig. 2C and D) and XPS spectra (Fig. 4). Construction of a p–n heterojunction between GQDs and the SnO_2_ surface further improves the charge transport properties and electrical properties. Oxygen functional groups (*e.g.*, C–O) in graphene induce p-type semiconducting properties due to the presence of oxygen atoms that tend to attract electrons.^46,47^ The formation of band bending at the interface between the n-type SnO_2_ and p-type GQDs enlarges the electron depletion layer, which enhances the modulation of electrical resistance under a NO_2_ gas flow. In addition, the electron transfer from the SnO_2_ surface to GQDs is highly efficient, as the SnO_2_ conduction band (CB) (4.5 eV)^48^ is near the lowest unoccupied molecular orbital (LUMO) level of GQDs (4.48 eV).^10^ As a result, our GQD@SnO_2_ nanodome gas sensor can be used to detect NO_2_ gas with high sensitivity and high selectivity and shows enhanced gas response over a broad operating temperature range, including room temperature.

## 4. Conclusions

In conclusion, the decoration of SnO_2_ with GQDs significantly enhances the NO_2_ gas response over a wide operating temperature range from room temperature to 150 °C. The nanodome structure of SnO_2_ improves the overall gas response due to its structural advances. The GQDs with discrete band gaps fabricated by the GIC method form a p–n heterojunction, in which electron transfer from n-type SnO_2_ to p-type GQDs enlarges the electron depletion layer on the surface, thereby resulting in effective resistance modulation. The GQD@SnO_2_ nanodome gas sensor shows enhanced NO_2_ gas sensing performance at room temperature based on the increased adsorption energy of NO_2_ gases by the oxygen functional groups on the GQDs. The GQD@SnO_2_ nanodome based gas sensor exhibits a response to 5 ppm NO_2_ gas (response = 4.8) at room temperature, while the pristine SnO_2_ nanodomes show no response. Furthermore, the response to NO_2_ is further improved with increasing operating temperature, with a 30 times higher response obtained at 150 °C compared to pristine SnO_2_ nanodomes. The GQD@SnO_2_ nanodome gas sensor also shows an extremely low detection limit (1.1 ppb) and high selectivity over various other gases. Our results clearly show the advantages of heterojunction formation of quantum-confined 0D materials with graphitic domains towards high-performance gas sensors. Subsequently, the investigation of other chemical functional groups for realizing a stable sensor response under humid conditions should be addressed for practical application of GQD-based room temperature gas sensors. The tunable electronic and chemical properties of GQDs present a possible strategy for the fabrication of room temperature gas sensors.

## Author contributions

Jinho Lee: investigation, data curation, writing – original draft. Minsu Park: investigation, data curation, writing – original draft. Young Geun Song: investigation, data curation. Donghwi Cho: investigation, methodology. Kwangjae Lee: methodology, resources. Young-Seok Shim: conceptualization, supervision, writing – review & editing. Seokwoo Jeon: conceptualization, resources, supervision, writing – review & editing.

## Conflicts of interest

There are no conflicts to declare.

## Supplementary Material

NA-005-D2NA00925K-s001
