# Supplementary material for: Role of graphene quantum dots with discrete band gaps on SnO2 nanodomes for NO2 gas sensors with an ultralow detection limit
Source: Nanoscale Adv. 2023 Apr 28;5(10):2767–75. doi: 10.1039/d2na00925k (PMC10186987; doi:10.1039/d2na00925k)
Supplement: NA-005-D2NA00925K-s001 [file NA-005-D2NA00925K-s001.pdf]

Electronic Supplementary Material (ESI) for Nanoscale Advances.
This journal is © The Royal Society of Chemistry 2023

Supplementary Information

Role of graphene quantum dots with discrete band gap on SnO_2 nanodomes for NO_2 gas sensors with an ultralow detection limit

Jinho Lee,[‡a] Minsu Park,[‡b] Young Geun Song,[c] Donghwi Cho,[d] Kwangjae Lee,[e] Young-Seok Shim,[*f] Seokwoo Jeon,[*a,g]

[a] *Department of Materials Science and Engineering, Korea Advanced Institute of Science and Technology (KAIST), Daejeon 34141, Republic of Korea*

[b] *Querrey Simpson Institute for Bioelectronics, Northwestern University, Evanston, IL 60208, USA*

[c] *Electronic Materials Research Center, Korea Institute of Science and Technology (KIST), Seoul 02791, Republic of Korea*

[d] *Thin Film Materials Research Center, Korea Research Institute of Chemical Technology, Yuseong, Daejeon 34114, Republic of Korea*

[e] *Department of Information Security Engineering, Sangmyung University, Cheonan 31066, Republic of Korea*

[f] *School of Energy, Materials and Chemical Engineering, Korea University of Technology and Education, Cheonan, 31253, Republic of Korea*

[g] *Department of Materials Science and Engineering, Korea University, Seoul, 02841, Republic of Korea*

* Corresponding authors.

E-mail addresses: ysshim@silla.ac.kr (Y.S. Shim), jeon39@kaist.ac.kr (S. Jeon).

‡ These authors contributed equally to this work.

Experimental section

Fabrication of GQD@SnO_2 nanodomes

Pt/Ti (thickness of 150 nm/30 nm) interdigitated electrode patterns (IDEs) consisting of 20 electrodes with 5 μm gap were fabricated on a SiO_2/Si substrate (thickness of 300 nm/550 μm). Nanodome-like structures were fabricated by the soft-templating method [1]. The polystyrene (PS) beads (700 nm, 5.0 wt%, Spherotech, USA) were dispersed in a water:ethanol = 1:1 (v:v) solution by a centrifuge process after the concentration reached 10 wt%. The PS bead solution was pipetted onto a glass slide positioned at an angle of 45° in a Petri dish with deionized water. The Pt/Ti IDEs patterned substrate and slide glass were treated by O_2-plasma treatment (CUTEMP, femtoscience) for 10 minutes before fabrication. The pipetted solution was dispersed onto the surface of deionized water and allowed to form a PS bead monolayer. The Pt/Ti IDEs patterned substrate were dipped into water and the PS bead monolayer was pulled out and dried at room temperature for 24 hours. SnO_2 was deposited onto the PS bead monolayer by using an electron-beam evaporator. A 150 nm thick SnO_2 layer was deposited at a rate of 1 Å s^{-1}. The deposited SnO_2 were annealed at 500 °C for 1 hour to simultaneously remove the PS templates and crystallize the SnO_2 nanodomes.

The GQDs were prepared from graphite intercalation compounds (GICs) through a previous method [2]. First, graphite and potassium sodium tartrate ($KNaC_4H_4O_6 \cdot 4H_2O$) were vigorously mixed at a ratio of 1:15 (w:w) and then ground. The mixture was heated in a heating mantle at 250 °C for 24 hours, which led to the formation of GICs. The as-prepared GICs were immersed in DI water and sonicated to exfoliate and cut the graphite. The crude GQD solution was filtered using centrifugal microfilters (10,000 NMWL, Amicon Ultra-15), followed by dialysis using a dialysis membrane for 3 days to remove any impurities and obtain pure GQDs <5 nm in size.

The GQD solution (0.1 mg ml^{-1}) was repeatably drop cast (total 100 μl) onto SnO_2 nanodomes and allowed to dry at room temperature for 24 hours.

Characterization and gas response measurements

The morphology of the GQD@SnO_2 nanodomes was investigated by field-emission scanning electron microscopy (FE-SEM, SU 5000, Hitachi). The structures and fast Fourier transform (FFT) images of GQDs were investigated by transmission electron microscopy (TEM, Tecnai F20, FEI Company). The crystallinity of the sensors was measured by X-ray diffraction (XRD, Ultima IV, RIGAKU) with a Cu-Kα radiation source (wavelength 1.5418 Å). The chemical bonding and binding energies of the sensor materials were investigated by X-ray photoelectron spectroscopy (XPS) using a K-alpha system (Thermo VG Scientific) with an Al-Kα X-ray source. The surface charging effect was corrected with C 1s peak at 284.7 eV as a reference. The Raman spectra were collected by Senterra system (Bruker) with 532 nm laser. The samples for XPS analysis and Raman analysis were prepared by annealing for 1 hour on a hot plate at room temperature, 50 °C, 100 °C, and 150 °C. The oxygen content in the GQDs was estimated using Auger electron spectroscopy (AES) with a source electron beam energy of >10 kV.

The responses of target gases were measured in a quartz tube with a 1-inch furnace (Lindberg, blue M). The operating temperature was controlled by a 1-inch furnace at room temperature, 50 °C, 100 °C, and 150 °C to evaluate the gas response mechanism at different operating temperatures. The gas flows were controlled to give a constant flow rate of 1000 sccm under dry condition (RH 0) using a mass-flow controller. The sensor resistance was measured using a Keithley 2401 instrument with a DC bias voltage of 0.5 V.

Supplementary Figures

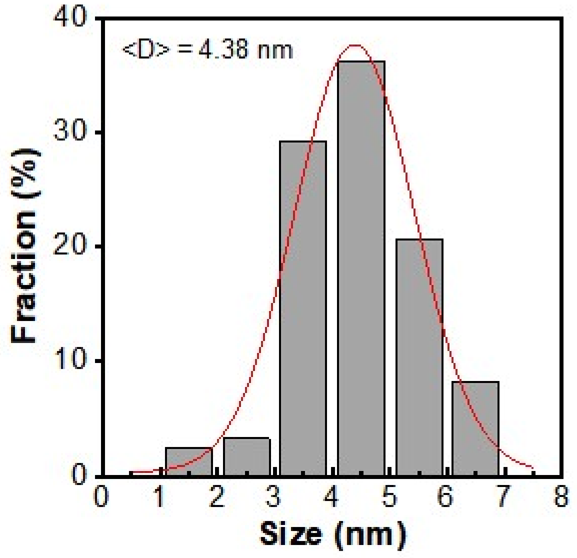

Fig. S1. Size distribution of pristine GQDs. <D> indicates average size.

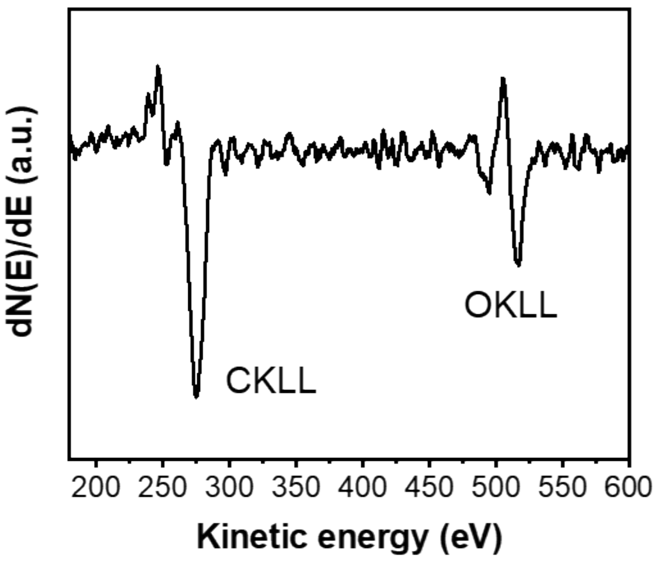

Fig. S2. Spectrum of magnified Auger electron spectroscopy (AES) for GQDs.

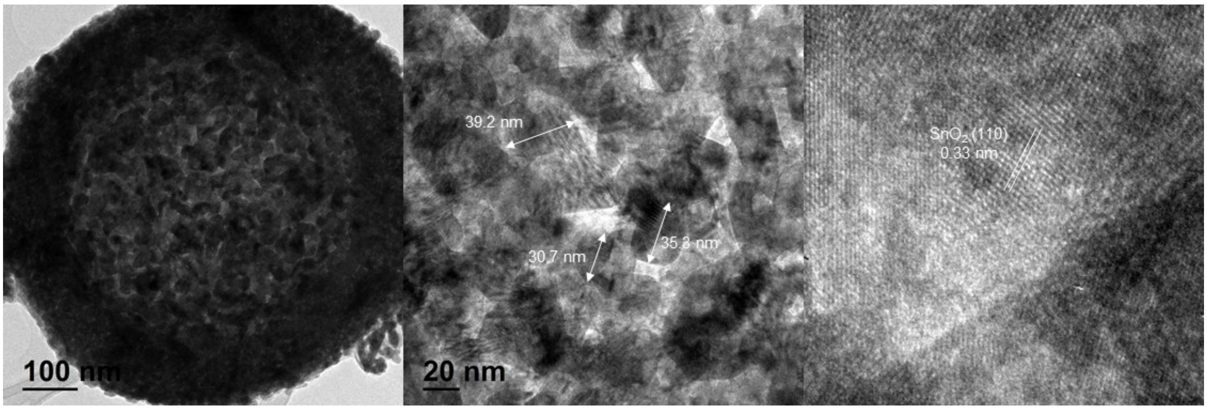

Fig. S3. HR-TEM image of individual SnO_2 nanodomes.

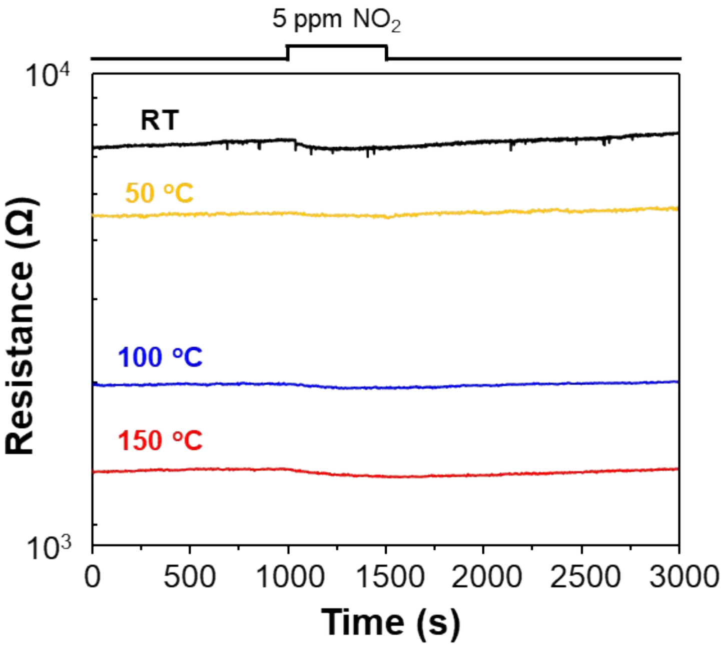

Fig. S4. Resistance curves for 5 ppm NO_2 as a function of operating temperature for a GQD@SnO_2 nanodomes under humid condition (relative humidity: 50 %).

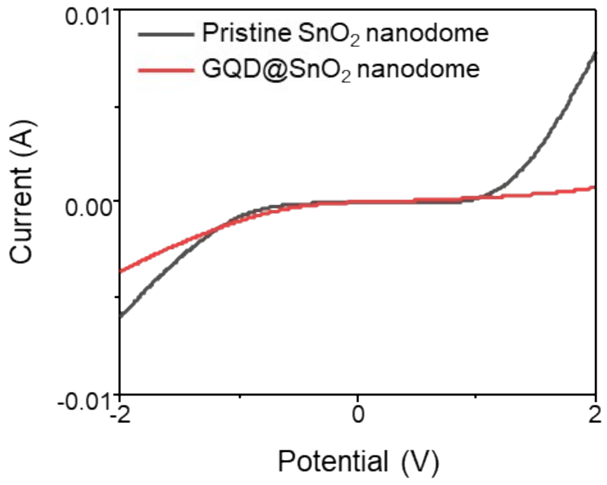

Fig. S5. *I-V* curve of SnO_2 nanodomes and GQD@SnO_2 nanodomes

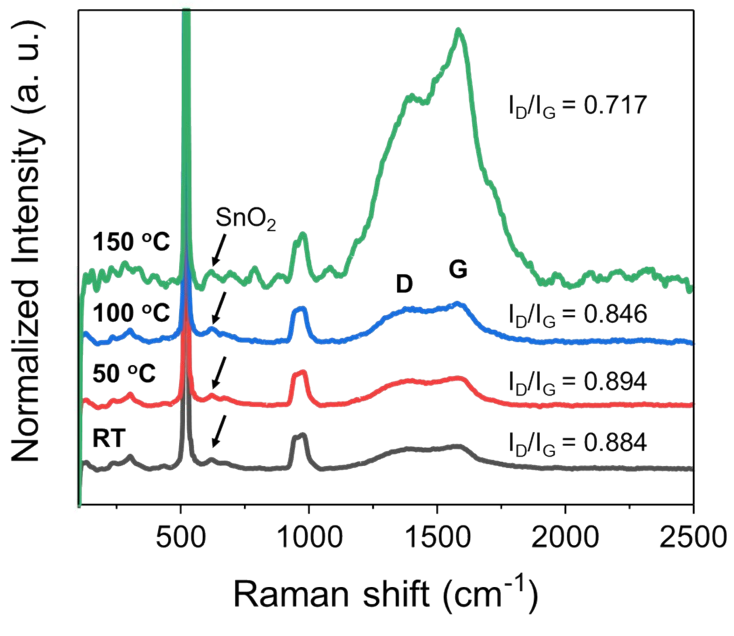

Fig. S6. Raman spectrum of the GQDs@SnO_2 nanodomes.

Table S1. Comparison of gas sensing performance of GQDs/graphene-based gas sensors.

Year	Material	Temp. (°C)	t_{res}[g]/t_{rec}[h] (s)	Response<br>($(R_a-R_g)/R_g$) or ($R_g-R_a)/R_a$)	LOD[i] (ppb)	Refs.
**2022**	GQD@SnO_2 nanodomes	150	322/105	39.1 (5 ppm)	1.1	*This work*
**2021**	N-GQDs[a]-SnO_2 hollow cube	130	59/33	417 (1 ppm)	-	3
**2021**	GQD-metal phthalocyanine hybrid	RT	100/100	15.8 (50 ppm)	50	4
**2020**	N-GD[b]-SnO_2-0D heterostructure	50	528/384	4336 (100 ppb)	20	5
**2020**	N-GQDs-3D ordered macroporous In_2O_3	100	95/36	81.7 (1 ppm)	100	6
**2020**	$BiVO_4/Cu_2O$/rGO[c]	60	51.3/87.5	8.1 (1 ppm)	-	7
**2020**	$CuWO_4$/rGO	RT	38/22	9.45 (50 ppm)	500	8
**2019**	rGO/ZnO-CT[d]	RT	140/630	1.15 (15 ppm)	43.5	9
**2018**	rGO-Co_3O_4	RT	90/2400	1.27 (5 ppm)	50	10
**2018**	CuO/rGO	RT	66/34	14 (1 ppm)	60	11
**2018**	WO_3/S-rGO[e]	RT	6/56	2.50 (20 ppm)	-	12
**2017**	SnO_2/N-RGO[f]	RT	45/168	1.38 (5 ppm)	-	13
**2017**	rGO-In_2O_3	RT	208/39	109 (1 ppm)	10	14
**2016**	ZnO/rGO	RT	75/132	2.19 (1 ppm)	50	15

[a] nitrogen-doped graphene quantum dots; [b] nitrogen-doped graphene dot; [c] reduced graphene oxide; [d] cotton thread; [e] sulfonated reduced graphene oxide; [f] nitrogen-doped reduced graphene oxide; [g] response time; [h] recovery time; [i] limit of detection

Table S2. Response/recovery times of bare SnO_2 and GQD@SnO_2 nanodome gas sensors at different operating temperatures.

	**Bare SnO_2 nanodome**		**GQD@SnO_2 nanodome**	
	Response time (s)	Recovery time (s)	Response time (s)	Recovery time (s)
**RT**	-	-	452	> 1500
**50 ^oC**	-	-	450	> 1500
**100 ^oC**	315	> 1500	459	1322
**150 ^oC**	59	1247	322	105

References

1. C. Zhang, S. Cvetanovic and J. M. Pearce, *MethodsX*, 2017, **4**, 229-242.
2. S. H. Song, M. H. Jang, J. Chung, S. H. Jin, B. H. Kim, S. H. Hur, S. Yoo, Y. H. Cho and S. Jeon, *Adv. Opt. Mater.*, 2014, **2**, 1016-1023.
3. Y. K. Lv, Y. Y. Li, H. C. Yao and Z. J. Li, *Sens. Actuators B Chem.*, 2021, **339**, 129882.
4. W. Jiang, X. Chen, T. Wang, B. Li, M. Zeng, J. Yang, N. Hu, Y. Su, Z. Zhou and Z. Yang, *RSC Adv.*, 2021, **11**, 5618-5628.
5. R. Purbia, Y. M. Kwon, H.-D. Kim, Y. S. Lee, H. Shin and J. M. Baik, *J. Mater. Chem. A*, 2020, **8**, 11734-11742.
6. Y.-K. Lv, Y.-Y. Li, R.-H. Zhou, Y.-P. Pan, H.-C. Yao and Z.-J. Li, *ACS Appl. Mater. Interfaces*, 2020, **12**, 34245-34253.
7. Q. Li, N. Han, K. Zhang, S. Bai, J. Guo, R. Luo, D. Li and A. Chen, *Sens. Actuators B Chem.*, 2020, **320**, 128284.
8. W. Zhang, D. Zhang and Y. Zhang, *J. Mater. Sci. Mater.*, 2020, **31**, 6706-6715.
9. W. Li, R. Chen, W. Qi, L. Cai, Y. Sun, M. Sun, C. Li, X. Yang, L. Xiang, D. Xie and T. Ren, *ACS Sensors*, 2019, **4**, 2809-2818.
10. B. Zhang, M. Cheng, G. Liu, Y. Gao, L. Zhao, S. Li, Y. Wang, F. Liu, X. Liang, T. Zhang and G. Lu, *Sens. Actuators B Chem.*, 2018, **263**, 387-399.
11. Z. Li, Y. Liu, D. Guo, J. Guo and Y. Su, *Sens. Actuators B Chem.*, 2018, **271**, 306-310.
12. T. Wang, J. Hao, S. Zheng, Q. Sun, D. Zhang and Y. Wang, *Nano Res.*, 2018, **11**, 791-803.
13. Z. Wang, C. Zhao, T. Han, Y. Zhang, S. Liu, T. Fei, G. Lu and T. Zhang, *Sens. Actuators B Chem.*, 2017, **242**, 269-279.
14. J. Liu, S. Li, B. Zhang, Y. Wang, Y. Gao, X. Liang, Y. Wang and G. Lu, *J. Colloid Interface Sci.*, 2017, **504**, 206-213.
15. Y. Xia, J. Wang, J.-L. Xu, X. Li, D. Xie, L. Xiang and S. Komarneni, *ACS Appl. Mater. Interfaces*, 2016, **8**, 35454-35463.
